# Increased brain size in mammals is associated with size variations in gene families with cell signalling, chemotaxis and immune-related functions

**DOI:** 10.1098/rspb.2013.2428

**Published:** 2014-01-22

**Authors:** Atahualpa Castillo-Morales, Jimena Monzón-Sandoval, Araxi O. Urrutia, Humberto Gutiérrez

**Affiliations:** 1 Department of Biology and Biochemistry, University of Bath, Bath BA2 7AY, UK; 2 School of Life Sciences, University of Lincoln, Lincoln LN6 7TS, UK

**Keywords:** encephalization index, brain evolution, gene expression

## Abstract

Genomic determinants underlying increased encephalization across mammalian lineages are unknown. Whole genome comparisons have revealed large and frequent changes in the size of gene families, and it has been proposed that these variations could play a major role in shaping morphological and physiological differences among species. Using a genome-wide comparative approach, we examined changes in gene family size (GFS) and degree of encephalization in 39 fully sequenced mammalian species and found a significant over-representation of GFS variations in line with increased encephalization in mammals. We found that this relationship is not accounted for by known correlates of brain size such as maximum lifespan or body size and is not explained by phylogenetic relatedness. Genes involved in chemotaxis, immune regulation and cell signalling-related functions are significantly over-represented among those gene families most highly correlated with encephalization. Genes within these families are prominently expressed in the human brain, particularly the cortex, and organized in co-expression modules that display distinct temporal patterns of expression in the developing cortex. Our results suggest that changes in GFS associated with encephalization represent an evolutionary response to the specific functional requirements underlying increased brain size in mammals.

## 1. Introduction

Mammalian species in general tend to have larger brain to body size ratios compared with other vertebrates and in some primate and cetacean species this relationship is particularly pronounced [1]. Large brains represent an evolutionarily costly adaptation as they are metabolically expensive, demand higher parental investment than in species with smaller brains and impose a substantial delay in reproductive age [1–5]. In spite of the cost and adaptive impact of larger brains, the precise nature of genomic changes accounting for variations in encephalization across mammalian species is at present poorly understood [6,7].

Whole-genome sequencing efforts have made it possible to study not just individual variations in specific sequences, but also large-scale differences in gene complements between species. Although overall gene number has changed little over the past 800 million years of metazoan evolution, comparative genomic studies have found large disparities among organisms in the number of copies of genes involved in a variety of cellular and developmental processes, and analyses of gene family evolution have shown that instances of gene family expansion and contraction are frequent [8–12]. In a recent analysis of *Drosophila* species, for instance, large numbers of gains and losses have been described, with over 40% of all gene families differing in size among the analysed species. Importantly, the fact that, in these species, rapid gene family size (GFS) evolution is accentuated in some functional categories strongly suggests that changes in gene number within gene families may reflect evolutionary responses to specific adaptive demands [10]. In this regard, gene duplication events specifically linked to distinct aspects of vertebrate evolution have been described. Examples include the expansion, during early evolution of the vertebrate lineage, of HOX and PAX gene families which are widely believed to have played a key part in the evolution of many known vertebrate innovations [13,14].

A major goal in evolutionary neurobiology is to understand the molecular changes underlying the extraordinary expansion in brain size observed in mammalian evolution. Whether changes in the number of copies of genes involved in distinct cellular and developmental functions has contributed to shaping the morphological, physiological and metabolic machinery supporting brain evolution in mammalian lineages is not known.

By conducting a genome-wide analysis of 39 fully sequenced mammalian species, we set out to establish whether changes in GFS can be linked to increased encephalization. Our results reveal a proportion of gene families displaying a positive association between GFS and level of encephalization significantly larger than expected by chance. This bias occurs most prominently in families associated with specific biological functions. By examining expression data in human tissues, we further found that gene families displaying the highest association between encephalization and GFS are also statistically enriched in genes that are prominently expressed in the brain, with maximal expression in the cortex and displaying an expression signature distinctly associated with cortical development.

## 2. Methods

### (a) Gene family annotations

Annotated gene families encompassing 39 fully sequenced mammalian genomes were obtained from Ensembl [15]. In the context of this annotation, a given gene family constitutes a group of related genes that include both paralogues within the same species and orthologues and paralogues from other species. Any given gene can only be assigned to a single gene family. GFS represents the total number of genes per gene family. In order to maximize the number of families covered in this study (more than 10 000), we included all gene families with members present in no less than six of the 39 mammalian species.

### (b) Encephalization index

Because larger species have larger brains, it is necessary to estimate brain mass controlling for the allometric effect of body size. We therefore adopted residuals of a log–log least-squares linear regression of brain mass against body mass as this is the most widely accepted index of encephalization (*Ei*; electronic supplementary material, table S1) [16,17]. While direct estimates of the ratio of brain mass to body mass have also been used as an alternative encephalization index [2,18], this measure is known to be poorly related to brain complexity across taxa [16,17]. Accurate estimates of brain residuals based on a sample of 493 mammalian species were kindly provided by Gonzalez-Lagos *et al.* [2].

### (c) Correlation coefficients of gene family size and encephalization index

Simple Pearson correlations between *Ei* and GFS as well as multiple regressions (where maximum lifespan (MLSP) was included as covariate, see below) were carried out using R-based statistical functions. Numerical randomizations to determine statistical significance were conducted using specially written R-based scripts.

### (d) Gene ontology terms analysis

Gene ontology (GO) annotations were obtained from the Gene Ontology database (www.geneontology.org). In this study, a particular GO term was associated with a family whenever that term was linked to any of its members in any species. Only terms found to be linked with more than 50 families were examined.

For each GO category, the average Pearson correlation coefficient was calculated. Statistical significance and expected average Pearson correlation per GO was measured using at least 10 000 equally sized random samples taken from the whole gene family population to directly determine the corresponding *p*-values. Bonferroni correction was used in all analyses to correct for multiple tests.

Enrichment analysis of GO categories was carried out by counting the number of families assigned to each GO term within the analysed set of gene families. However, any bias in family counts per GO within a set of families could be owing to a bias in the overall density of GO annotation events within that sample. In order to adjust for differences in the density of GO annotations between the test and background samples, we divided the family counts per GO from each sample, by the samples' average number of GO annotations per family. Statistical significance was numerically assessed by obtaining the expected (adjusted) number of families per GO in 10 000 equally sized random samples derived from the overall population of gene families.

### (e) Maximum lifespan and partial correlation coefficients

MLSP recorded for each species was obtained from the animal ageing and longevity database (AnAge) [19]. To correct for the potential contribution of MLSP to the association between GFS and *Ei*, partial correlation coefficients were calculated for each gene family, including MLSP as covariate. The resulting partial coefficient represents the contribution of *Ei* to the variance in GFS which is not explained by variations in MLSP. Only those gene families displaying a significant partial correlation coefficient (*p* < 0.01) between GFS and *Ei* were considered further.

### (f) Phylogenetic relatedness test

Phylogenetic generalized least-square approach (PGLS) and maximum-likelihood estimation of *λ*-values were carried out using the CAPER module in R. Because the parameter *λ* measures the degree to which the phylogeny predicts the pattern of covariance of a given trait across species (where *λ*-values close to 0 represent no phylogenetic autocorrelation while values close to 1 represent full phylogenetic autocorrelation) [20–22], this approach allows us to obtain a single accurate measure of phylogenetic autocorrelation for each individual gene family. In order to remove the effect of phylogenetic relationships from our analysis, we determined the parameter *λ* for each of the 713 gene families with significant partial correlation coefficients for *Ei* and GFS (correcting for MLSP) and eliminated all gene families with a significant phylogenetic interdependence (*p* < 0.05 of *λ* = 0, and *p* > 0.05 of *λ* = 1). This filtering resulted in 501 gene families on which GO enrichment analyses were subsequently carried out as described above.

### (g) Gene expression in human brain

RNA-seq data were obtained for 18 052 genes in a total of 16 human tissues, including brain, derived from the Illumina human body map dataset (Ensembl v. 62). Individual genes were categorized as *prominently expressed* in the brain if their expression level in this tissue was the highest or second highest among all 16 tissues included (top 12.5th percentile). Over-representation was assessed by counting the number of these genes within a given sample. Statistical significance was assessed by comparing this count with those observed in 10 000 equally sized random samples drawn from the wider pool of gene families.

### (h) Co-expression network analysis

Weighted gene co-expression network analysis was carried out based on pairwise Pearson correlations between the expression profiles obtained from the BrainSpan database (http://www.brainspan.org) for over 21 000 genes. Unsupervised hierarchical clustering was used to detect groups, or modules, of highly co-expressed genes following the method described by Zhang & Horvath [23].

## 3. Results

### (a) Gene family size variations in line with encephalization are over-represented in mammals

In order to assess the relationship between encephalization and GFS variations in mammalian taxa, gene family annotations for 39 fully sequenced mammalian genomes were obtained from Ensembl [15]. We included in this study all families with members present in no less than six of the 39 mammalian species (see Methods). This resulted in a total of 12 373 non-overlapping gene families encompassing 595 535 genes, with a mean number of 48.13, and a number of copies per gene family per species ranging from 0 to 448.

**Figure 1.**
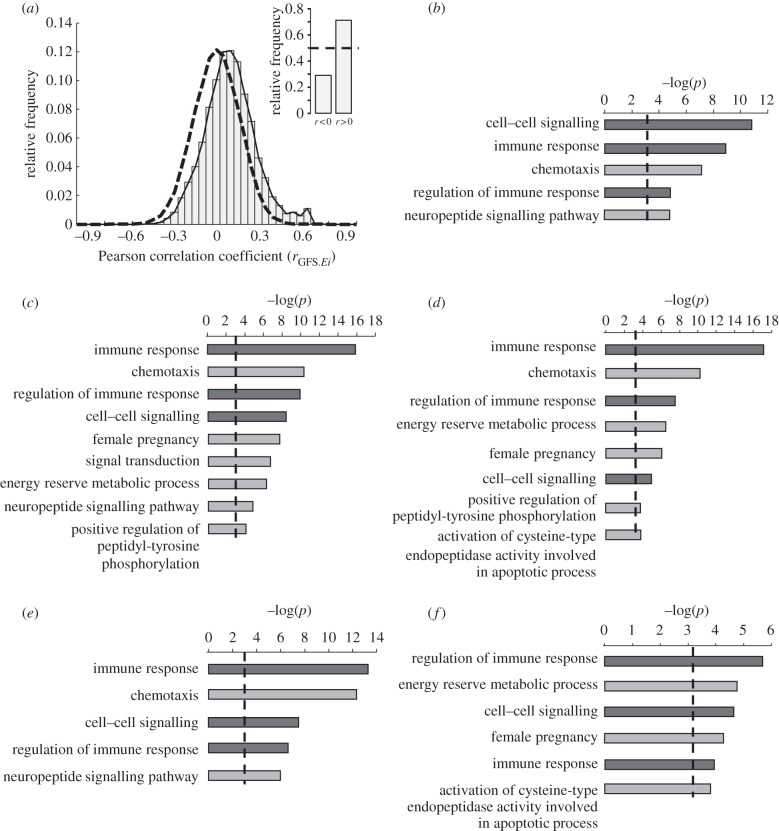
Enrichment of gene family size variations in line with increased encephalization in mammals. (*a*) Histogram showing the distribution of correlation coefficients for GFS and *Ei* in 12 373 gene families encompassing 39 mammalian genomes. A randomization-based estimation of the expected distribution is represented by the dashed line. Inset: distribution of positive and negative correlations relative to the expected distribution (dashed line). (*b*) Deviations from random expectations in the mean correlation coefficient of gene families associated with individual GO terms (expressed as −log(*p*-value)). Only GO categories with a significant bias are shown. (*c*) Over-representation of GO terms among gene families most significantly associated with encephalization (*p* < 0.05, *n* = 1292). (*d*) GO enrichment analysis among the families displaying the most significant correlation with encephalization after removing all families with a stronger association with MLSP than with *Ei* (*n* = 927). (*e*) GO-terms enrichment analysis among gene families with the most significant positive partial correlation coefficients for *Ei* after controlling for the contribution of MLSP in a multiple regression analysis (*n* = 713). (*f*) GO-terms enrichment analysis among gene families with the most significant positive partial correlation coefficients for *Ei* with no significant phylogenetic interdependence (*n* = 501). Bonferroni-corrected significance thresholds are indicated with a dashed line. Dark bars indicate common GO terms across all five analyses.

*Ei* for each species was defined as the residual of a log–log least-squares linear regression of brain mass against body mass (see Methods). We obtained correlation coefficients for GFS and *Ei* for each gene family and the resulting distribution of correlation coefficients showed a distinct shift towards positive values (figure 1*a*). A Monte Carlo simulation of the expected distribution based on random permutations of GFS values across species revealed that the observed bias is highly significant (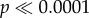). In total, we found 8789 families with *r* > 0, representing a shift of 2602 gene families from the negative to the positive tail of the distribution relative to the expected equal number of positively and negatively correlated families (*χ*^2^ = 2189.608, *p* ≈ 0; figure 1*a*, inset). This result demonstrates a highly pronounced over-representation of gene families displaying a positive association between GFS and *Ei*. This observation is not explained by an overall expansion in gene number across species in line with *Ei* (*r* = 0.251, *p* = 0.127), but rather by an over-representation of small gene families among those highly associated with encephalization, combined with few larger gene families displaying decreases in size.

We next asked whether the observed enrichment of *Ei*-related GFS variations was unspecific in terms of the gene populations involved or, alternatively, if this enrichment occurred in gene families specifically associated with certain biological functions. To this end, we used functional GO annotations for ‘biological processes’ and carried out two complementary tests to assess deviations (from random expectations) in the distribution of GO terms associated with gene families displaying a high correlation between GFS and *Ei*. First, we examined whether there were any significant deviations in the mean correlation coefficient of gene families associated with individual GO terms (see Methods). Out of all 260 functional categories included, only gene families associated with cell–cell signalling, immune response, chemotaxis, neuropeptide signalling pathways and regulation of immune response displayed a significantly higher than expected average correlation values, between GFS and *Ei*, after Bonferroni correction (figure 1*b*). By contrast, no significant bias was observed in functional categories containing families with negative average correlations (not shown).

Second, we measured over-representation of GO terms among the gene families whose GFS variations were most significantly associated with *Ei* (*r* > 0, *p* < 0.05, *n* = 1292). Among these families, we found that GO terms for immune response, chemotaxis, regulation of immune response, female pregnancy, cell–cell signalling, signal transduction, energy reserve metabolic processes, positive regulation of peptidyl-tyrosine phosphorylation and neuropeptide signalling pathways were significantly over-represented after Bonferroni correction (figure 1*c*). No GO terms were found to be significantly over-represented among gene families with the highest negative covariance between GFS and *Ei* (not shown). Taken together, these results show that the observed collective variation in GFS in line with encephalization is not randomly distributed across functional categories but is significantly pronounced in families associated with specific biological functions.

### (b) Association between gene family size and encephalization is not explained by lifespan variations

A number of studies on brain evolution have uncovered a robust relationship between relative brain size and lifespan [2,24,25]. In agreement with this, we found a strong association between MLSP and *Ei* among the species included in this study (*r* = 0.7912, *p* < 10^−8^). Thus, the observed associations between *Ei* and GFS could be secondary to an underlying association between MLSP and GFS. Of the 1292 most significantly correlated families (*r* > 0, *p* < 0.05), 927 displayed a stronger association with *Ei* than with MLSP (*r*_(*Ei*, GFS)_ > *r*_(MLSP, GFS)_), thereby suggesting a preferential contribution of *Ei* to the observed bias in the correlation distribution (*χ*^2^ = 858.74, *p* = 3.3572e^−187^, relative to a random equal distribution of stronger associations). GO enrichment analysis was then repeated including only these 927 families revealing a significant over-representation of gene families associated with immune response, chemotaxis, regulation of immune response, energy reserve metabolic processes, female pregnancy, cell–cell signalling, positive regulation of peptidyl-tyrosine phosphorylation and activation of cysteine-type endopeptidase activity involved in apoptotic processes (figure 1*d*). It is worth noting that the complementary GO enrichment analysis carried out on gene families with both the most significant association between MLSP and GFS (*r* > 0, *p* < 0.05) and a stronger association with MLSP than *Ei* (*r*_(MLSP, GFS)_ > *r*_(*Ei*, GFS)_, *n* = 1321), resulted in no significant enrichment of any GO category. These results shows that enrichment of specific GO terms occurred only among gene families preferentially associated with degree of encephalization, whereas GFS variations potentially associated with increased MLSP showed no significant association with any particular functional category.

Because MLSP may still partly explain the covariance between GFS and *Ei* even if the correlation coefficient of GFS with *Ei* is higher than with MLSP, we used multiple regression analysis to obtain partial correlation coefficients between GFS and *Ei* after controlling for the contribution of MLSP (see Methods). GO terms enrichment analysis was then carried out only among those gene families with the most significant positive partial correlation coefficients (partial *r* > 0, *p* < 0.05, *n* = 713). This analysis revealed a significant enrichment of families functionally associated with regulation of immune response, chemotaxis, cell–cell signalling and neuropeptide signalling pathways (figure 1*e*). These results show that variations in GFS specifically associated with encephalization (i.e. not accounted for by variations in MLSP) are also specifically associated with distinct biological functions.

### (c) Phylogenetic relatedness does not explain the observed bias in the distribution of gene families associated with encephalization

For a given gene family, any association between *Ei* and GFS could be the secondary to existing phylogenetic relationships among the species analysed, as in the absence of any selective forces, closely related species will tend to have both similar degrees of *Ei* and similar GFS [20,22]. In order to determine the degree to which phylogenetic effects contribute to the observed shift in the correlation distribution, we used a PGLS approach (see Methods) [20,22]. Out of 713 gene families with the most significant positive partial correlation coefficients between *Ei* and GFS (after correcting for MLSP, see previous analysis), we found a total of 501 gene families for which phylogenetic relationships among species could not account for the covariance between GFS and *Ei*. Among these families, we observed a significant over-representation of gene families associated with regulation of immune response, cell–cell signalling, energy reserve metabolic processes, female pregnancy and activation of endopeptidase activity involved in apoptosis (figure 1*f*). These findings demonstrate that the over-representation of specific biological functions among those gene families most strongly associated with higher *Ei* is neither explained by the known association between MLSP and *Ei* nor by existing phylogenetic relationships among the species analysed.

### (d) Gene families with size increases in line with encephalization show expression signatures consistent with brain functions

**Figure 2.**
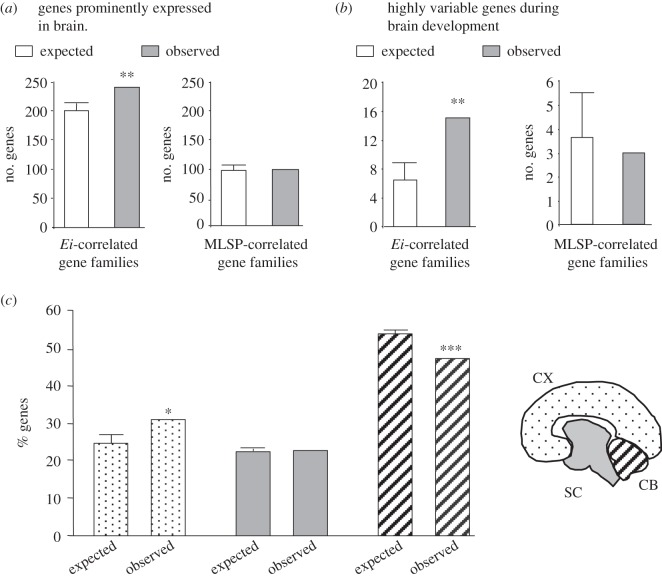
Relationship between *Ei*-associated GFS variations and patterns of gene expression in the human nervous system. (*a*) Over-representation of genes prominently expressed in the human brain (top 12.5th percentile) among the top 501 *Ei* -associated or the top MLSP-associated gene families compared to random expectations. (*b*) Over-representation of genes displaying the highest expression variance during human cortical development relative to adulthood among the top *Ei*-associated or the top MLSP-associated gene families. (*c*) Percentage of genes maximally expressed in the cortical (CX), subcortical (SC) and cerebellar (CB) regions respectively. Expected values (mean ± s.e.m.) were numerically determined using sized-matched random samples of genes drawn from the wider pool of gene families. *p < 0.01; **p < 0.001; ***p < 0.0001.

To assess whether gene family variations in line with encephalization were directly associated with brain function, we characterized the potential relationship between *Ei*-associated GFS variations and patterns of gene expression in the human nervous system. For this analysis, we selected the top 501 *Ei*-associated gene families with both the most significant partial correlation coefficient between *Ei* and GFS and no significant phylogenetic effects (figure 1*f*). Using available expression data from the Illumina human body map (see Methods), we looked at the possible over-representation of genes highly expressed in the human brain within the selected 501 gene families. Individual genes were categorized as *prominently expressed* in the brain if their expression level in this tissue was the highest or second highest among all 16 tissues included (top 12.5th percentile). Statistical significance was assessed by comparing with equally sized random samples drawn from the wider pool of gene families (see Methods). This analysis revealed a significant enrichment, within these gene families, of genes prominently expressed in the brain (figure 2*a*). By contrast, no significant enrichment of genes prominently expressed in the brain was detected among those gene families with the strongest association with MLSP and no significant phylogenetic effects (figure 2*a*).

Genes involved in cortical development have been shown to display higher variance in expression level during the developmental period of the cerebral cortex compared with adulthood [26]. We therefore looked at the possible representation of genes displaying the highest expression variance during human cortical development relative to adulthood, as defined by Sterner *et al*. [26], within the same 501 gene families and found a significant enrichment of genes displaying this pattern of expression (figure 2*b*). By contrast, no significant enrichment of these same genes was observed among the top MLSP-associated gene families (figure 2*b*).

We next asked whether there was any statistical bias in the relative expression of *Ei*-associated gene families across different brain regions. Using human brain RNA-seq data from the BrainSpan dataset (see Methods), we obtained the average expression for each gene in the cortex, subcortical regions or cerebellum and split them into three categories according to the region where the highest average expression was found. This analysis revealed a statistically significant enrichment, among those genes contained within the top 501 *Ei*-correlated gene families, of genes maximally expressed in the cortex (figure 2*c*). No significant enrichment of genes maximally expressed in subcortical regions was observed among these families. By contrast, genes maximally expressed in the cerebellum were found to be significantly under-represented among the top *Ei*-correlated gene families. Taken together, these results reveal that gene families displaying the highest association between *Ei* and GFS are enriched in genes that are prominently expressed in the brain, with maximal expression in the cortex and display an expression signature distinctly associated with cortical development.

**Figure 3.**
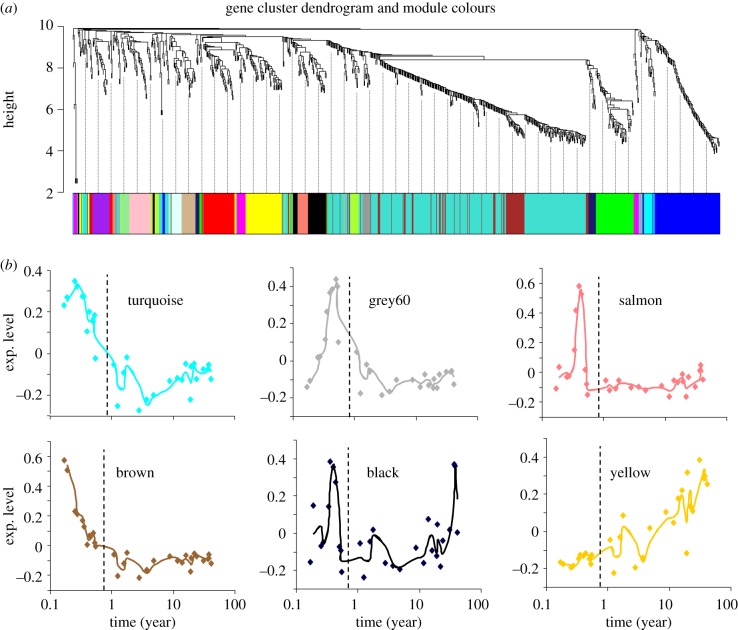
Temporal patterns of cortical expression of *Ei*-associated gene families. (*a*) Weighted gene co-expression network analysis was used to detect co-expression modules among genes contained within the top 501 *Ei*-associated gene families using human brain temporal expression data, revealing 18 co-expression modules (coloured). (*b*) Developmental time course of expression of six representative modules summarized by the level of expression of the eigengene associated with each module's co-expression matrix. Birth point is indicated with a dashed line.

In order to characterize further the cortical expression profile of *Ei*-associated gene families, we used a weighted gene co-expression network analysis approach to identify modules of co-expression among genes contained within the top 501 *Ei*-correlated gene families. Using human developmental expression data derived from the BrainSpan dataset, we identified 18 modules (figure 3*a*) associated with distinct temporal patterns of expression. Figure 3*b* shows the time course of expression of six of these modules summarized by the eigengene associated with each module's co-expression matrix. Some of these modules showed the highest expression levels during the early or late fetal period followed by a progressive decline in expression levels with age. This trend may reverse in some instances in late-adult stages (black module, figure 3*b*) or show a progressive increase throughout development as illustrated by the yellow module.

## 4. Discussion

Our results reveal a highly significant over-representation of gene families displaying a positive association between GFS and level of encephalization. This bias occurs most prominently in families associated with specific biological functions. The most robust and consistent bias was observed in gene families associated with cell signalling, immune regulation and chemotaxis.

While chemotaxis and cell signalling functions are known to play central roles in the nervous system, the significance of the observed enrichment of immune system-associated functions among gene families displaying the highest association between GFS and *Ei* is less clear. In recent years, however, signalling and regulatory mechanisms originally described in the immune system have increasingly been found implicated in key neural-specific roles both in the developing and adult nervous system [27–33]. In addition, in the human cerebral cortex, immune system-related functions have been found to be significantly over-represented among genes displaying higher expression variability in the developing cerebral cortex than in the adult [26], suggesting a substantial involvement of immune-related signals during cortical development.

Our results, showing a significant over-representation of immune-related functions among *Ei*-associated gene families, support the notion of an underlying and substantial overlap in the regulatory and signalling machinery shared by both the immune and nervous system and in particular during development of the latter.

One possible interpretation is that the observed enrichment of immune-related functions among *Ei*-associated gene families reflects an underlying expansion of immune surveillance in mammals that could be in some way permissive to increased encephalization. While we cannot rule out this possibility, at present, there is little evidence in support of any systematically pronounced and sustained expansion of immune functionalities in mammalian lineages [34]. An alternative interpretation is that signalling and regulatory molecular components that were originally involved in immune-specific functions became gradually recruited by the nervous system in response to the developmental and functional demands of increasingly more complex brains.

The observed association between degree of encephalization and variations of GFS in a large number of gene families is further supported by our finding that *Ei*-associated gene families display a transcriptional signature consistent with brain-specific functions. Indeed, among the gene families most highly correlated with encephalization with no significant phylogenetic effects, we found a statistically significant enrichment of genes prominently expressed in the brain, strongly indicating that these genes are under comparably higher demand in the nervous system relative to other tissues. When restricting the analysis to the relative expression levels within central nervous system regions, we found that these families are enriched in genes prominently expressed in the cortex, suggesting that *Ei*-correlated changes in GFS may have played a substantial role supporting key aspects of cortical evolution. In this regard, it is worth noting that brain evolution in mammalian lineages is characterized by a disproportional expansion of the brain cortex [35,36]. Analysis of the developmental pattern of expression of these families in the human cortex showed that these genes are organized in co-expression clusters or modules with distinct temporal profiles suggesting a substantial involvement of these families in the developmental organization of the brain.

Genes with the highest degree of connectivity within a module are termed hub genes and are expected to be functionally important within the module. By way of illustration, we examined the turquoise module (figure 3*b*) and identified a member of a zinc finger gene family (gene family ID: ENSFM00620000999432) as its main hub gene. Interestingly, all but two of the 20 members of this gene family in humans are contained within the same co-expression module. Because genes contained within a co-expression module are thought to be functionally related [23,37], the fact that most members of this zinc finger family are found within the same co-expression module strongly suggests that these genes are functionally related during brain development. We reconstructed the phylogenetic tree of this family and found that the observed pattern is the result of a combination of events of gene loss and gene gain from an original set of four ancestral proteins at the base of the mammalian evolution, overall resulting in a steady increase in the number of gene family members in line with increased level of encephalization (*r* = 0.7547, *p* = 2.86 × 10^−8^).

## 5. Conclusion

In this study, we have found a significant over-representation of GFS variations in line with increased encephalization in mammals. Importantly, this relationship is not accounted for by known correlates of brain size and is not explained by phylogenetic relatedness. The observed bias occurs most prominently in families preferentially expressed in the brain, in particular the cortex, and significantly associated with distinct biological functions.

Based on our results, we propose that variations in GFS associated with encephalization provided an evolutionary support for the specific cellular, physiological and developmental demands associated with increased brain size in mammals.
